# Sepsis: the evolution of molecular pathogenesis concepts and clinical management

**DOI:** 10.1002/mco2.70109

**Published:** 2025-02-23

**Authors:** Zhongxue Feng, Lijun Wang, Jing Yang, Tingting Li, Xuelian Liao, Yan Kang, Fei Xiao, Wei Zhang

**Affiliations:** ^1^ Institute of Critical Care Medicine, State Key Laboratory of Biotherapy and Cancer Center West China Hospital, Sichuan University Chengdu Sichuan China; ^2^ Department of Critical Care Medicine West China Hospital, Sichuan University Chengdu Sichuan China; ^3^ Department of Intensive Care Unit of Gynecology and Obstetrics West China Second University Hospital, Sichuan University Chengdu Sichuan China

**Keywords:** anti‐inflammatory drugs, clinical trials, host defenses, hyperinflammation, immunoenhancing drugs, immunosuppression, sepsis

## Abstract

The mortality rate of sepsis is approximately 22.5%, accounting for 19.7% of the total global mortality. Since Lewis Thomas proposed in 1972 that “it is our response that makes the disease (sepsis)” rather than the invading microorganisms, numerous drugs have been developed to suppress the “overwhelming” inflammatory response, but none of them has achieved the desired effect. Continued failure has led investigators to question whether deaths in septic patients are indeed caused by uncontrolled inflammation. Here, we review the history of clinical trials based on evolving concepts of sepsis pathogenesis over the past half century, summarize the factors that led to the failure of these historical drugs and the prerequisites for the success of future drugs, and propose the basic principles of preclinical research to ensure successful clinical translation. The strategy of targeting inflammatory factors are like attempting to eliminate invaders by suppressing the host's armed forces, which is logically untenable. Sepsis may not be that complex; rather, sepsis may be the result of a failure to fight microbes when the force of an invading pathogen overwhelms our defenses. Thus, strengthening the body's defense forces instead of suppressing them may be the correct strategy to overcome sepsis.

## Graphical abstract

(A) The innate and adaptive immune responses established during long‐term evolution can eliminate most invading microorganisms. (B) Sepsis only occurs when the balance is disrupted and the pathogen force overwhelms the host's defense force. (C) Only when the superposition of external forces (sensitive antimicrobial drugs) and internal forces (activation of the host immunity) overpowers invading pathogens can sepsis be cured.

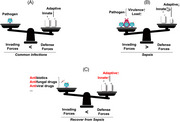

## 1 INTRODUCTION

**FIGURE 1:**
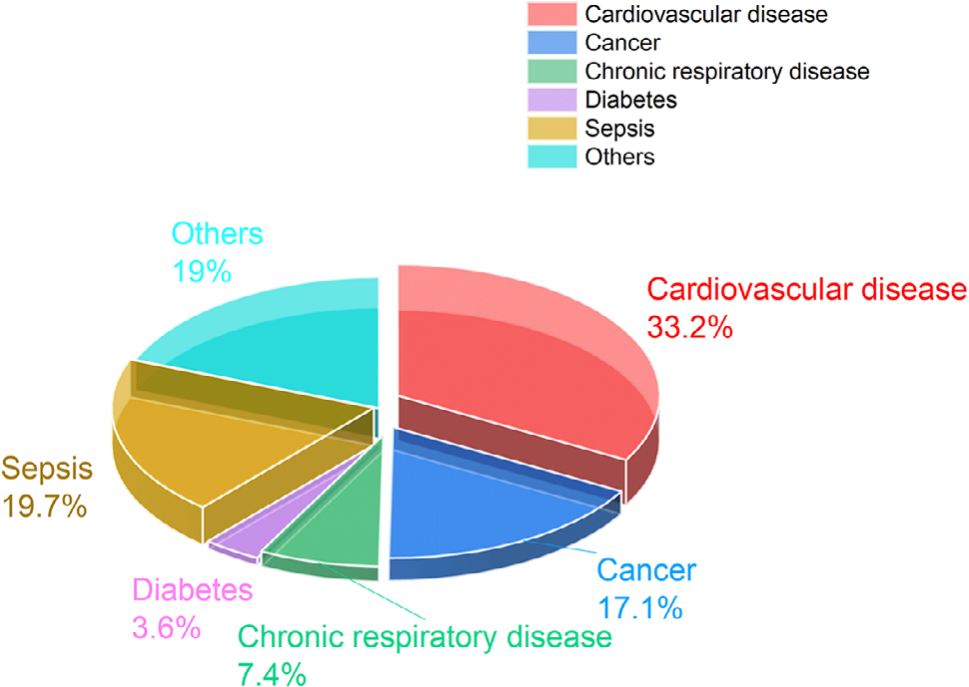
Deaths due to different causes worldwide. According to data provided by the World Health Organization (WHO) (htps://www.who.int/publications/i/item/9789240074323), sepsis accounts for 19.7% of deaths, exceeding the number of deaths from cancer and becoming the second leading cause of death.

According to the Third International Consensus Definition of Sepsis and Septic Shock (Sepsis‐3), sepsis is defined as life‐threatening organ dysfunction caused by a dysregulated host response to infection.^1^, ^2^, ^3^, ^4^, ^5^, ^6^ Rudd et al.^7^ calculated sepsis‐related mortality from multiple cause‐of‐death data from 109 million individual death records: 48.9 million cases of sepsis were recorded worldwide, of which 11 million resulted in sepsis‐related deaths, accounting for 19.7% of all deaths worldwide (Figure 1). Buchman et al.^8^ reported that hospitalization costs for Medicare beneficiaries increased from $17,792,657,303 in 2012 to $22,439,794,212 in 2018. The latest epidemiological study on sepsis in China, performed in 2023, revealed that from 2017 to 2019, sepsis‐related deaths accounted for nearly 13.1% of all deaths.^9^ Epidemiological and health economic analyses have shown that sepsis has become a serious public health problem and has caused a considerable economic burden. However, despite years of basic research and clinical trials, the mortality rate of sepsis is still higher than 20%.^7^

The paucity of effective sepsis‐specific treatments is not due to a lack of effort. In 1972, Lewis Thomas proposed in the *New England Journal of Medicine* that “it is our response that makes the disease (sepsis),” suggesting that patients with sepsis are more endangered by their own responses than by invading microorganisms.^10^ Since then, therapies that target inflammatory mediators have dominated sepsis research for approximately half a century. According to the prevailing theory that sepsis is caused by an overactive inflammatory response to infection, numerous drugs have been developed to block the inflammatory reaction, including antiendotoxin antibodies, antitumor necrosis factor α (TNF‐α) antibodies, corticosteroids, and other drugs that target inflammatory mediators.

Between 1995 and 2020, 1316 adult sepsis‐related clinical trials, in which adolescents and children were excluded, were registered at clinicaltrials.gov.^11^ According to data from Chictr.org.cn and clinicaltrials.gov, 683 sepsis‐related clinical trials have been registered in China. In these efforts, effective treatments that can reduce mortality generally include life‐supportive care (including fluid resuscitation, renal replacement therapy, mechanical ventilation, extracorporeal membrane oxygenation, etc.) and antimicrobial therapy that directly targets the causative pathogen. However, clinical trials of agents that target inflammatory mediators, the putative pathogenesis of sepsis, have not yielded the desired improvement in mortality rates among sepsis patients,^12^, ^13^ which has led researchers to question whether deaths in septic patients are indeed caused by uncontrolled inflammation.

Here, we review the history of the development of drugs based on the evolving concepts of sepsis. We first review the evolving definition and pathogenesis of sepsis. Then, we review the clinical trials of drugs designed on the basis of these evolving concepts. Finally, we summarize factors that led to the failure of these historical drugs and the prerequisites for the success of future drugs, hoping to provide new perspectives for discovering new therapies for sepsis. We narrow the scope of this review to drugs that have entered clinical trials and mention only briefly the therapeutic strategies that are still in the basic research stage, such as vascular repair and the regulation of mitochondrial function and metabolism.

An analysis of the evolving concepts and treatments of sepsis suggests that an excessive inflammatory response can also be considered, as even if the innate immune response is mobilized to its full potential, the host is unable to eliminate the invading pathogen—this reflects a fierce battle and should not be understood as “it is our own response that causes the disease.” Sepsis may simply be a result of a failing battle against microorganisms when the force of the invading pathogen overpowers the body's own defenses. Thus, strengthening external forces (accurate pathogen identification and sensitive antimicrobial drugs) and internal forces (strengthening adaptive immunity and fine‐tuning innate immunity) may be the right way to overcome sepsis.

## 2 THE EVOLVING CONCEPTS OF SEPSIS

Here, we briefly review the evolving definition and pathogenesis of sepsis. The design of drugs for sepsis treatment is based on these different concepts of the pathogenesis of sepsis in different historical periods.

### 2.1 The evolving definition of sepsis

The term “sepsis” derives from the ancient Greek word “σήψη (sepo),” which means decay or putrefaction.^14^, ^15^ The word “sepsis” first appeared in Homer's poems more than 2700 years ago. The term also appears in Corpus Hippocraticum, a work by the great physician and philosopher Hippocrates in 400 BC.^15^ These findings suggest that sepsis is a dangerous, odiferous, biological decay that occurs in the body.

The modern definition of sepsis was officially proposed in 1991. The American College of Chest Physicians (ACCP) and the Society of Critical Care Medicine (SCCM) convened in Chicago in 1991 and developed the first modern definition of sepsis as systemic inflammatory response syndrome (SIRS) caused by infection (Sepsis‐1).^16^ However, the diagnostic criteria for SIRS were too broad, resulting in high sensitivity but low specificity. This low specificity led to the misdiagnosis of many patients who did not have sepsis, thereby reducing the effectiveness of the definition.

In 2001, ACCP, SCCM, the European Society of Intensive Care Medicine, the American Thoracic Society, and the Surgical Infection Society held the second consensus meeting (Sepsis‐2), and the definitions of sepsis and septic shock were revised to include thresholds for organ damage. Compared with the criteria established at the 1991 consensus conference, the signs and symptoms of sepsis agreed upon at Sepsis‐2 were much more numerous and detailed.^17^ Sepsis was divided into four different subtypes: SIRS, sepsis (SIRS + infection), severe sepsis (sepsis + multiple organ dysfunction syndrome), and septic shock (sepsis + shock).

**FIGURE 2:**
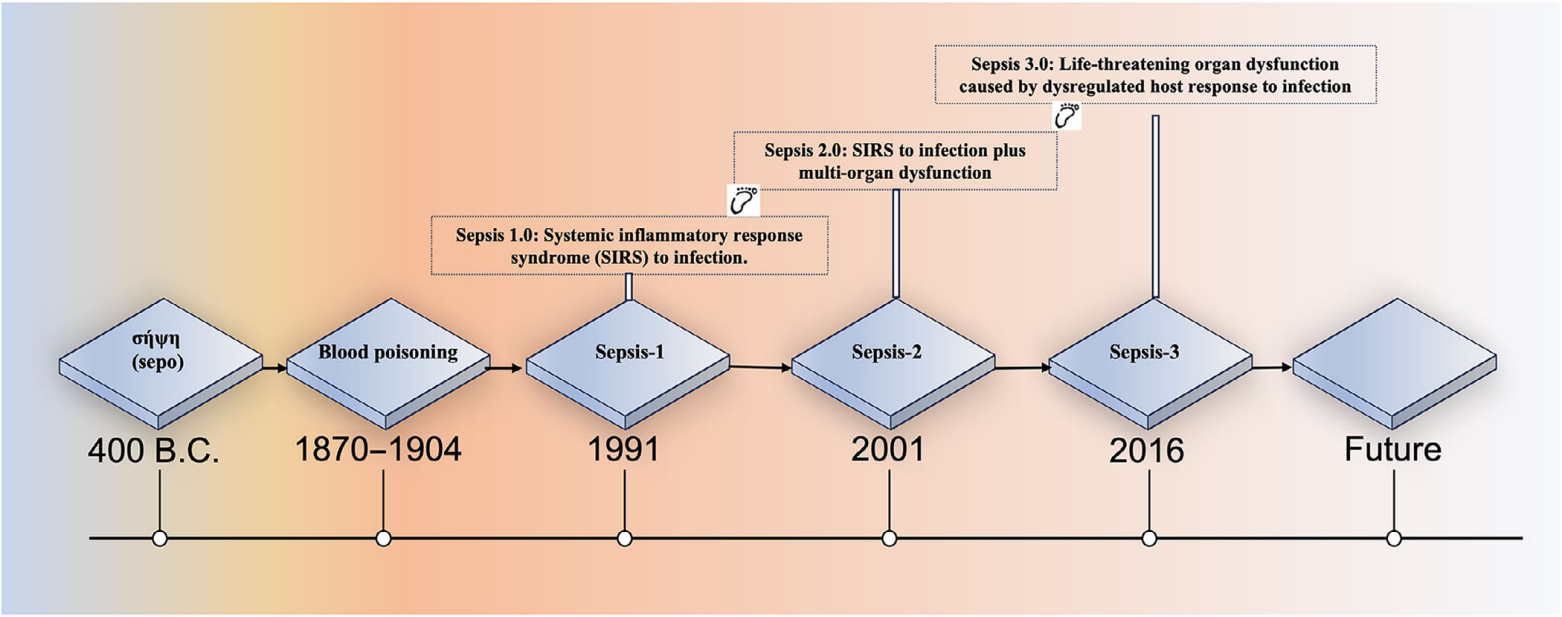
The evolving definition of sepsis. The term "sepsis" originates from the acient Greek word "sepo", meaning decay. From the 19th to 20th century, it was considered a form of "blood poisoning". The modern definition of sepsis was officially proposed in Sepsis‐1 in 1991 as "systemic inflammatory response syndrome (SIRS) caused by infection". However, its broad diagnostic criteria led to low specificity and misdiagnosis. In 2001, Sepsis‐2 refined the definition, adding organ damage thresholds and categorizing sepsis into 4 subtypes. By 2016, advancements necessitated Sepsis‐3, which refined sepsis as "life‐thereatening organ dysfunction caused by dysregulated host response".

New advances and insights into the epidemiology and treatment of sepsis over the 15 years from 2001 to 2016 necessitated a new definition of sepsis. In addition, the inclusion of 21 specific diagnostic criteria made the Sepsis‐2 definition overly complex and challenging to implement in the fast‐paced environment of clinical practice. This complexity limited the adoption and clinical applicability of the Sepsis‐2 definition. Therefore, in 2016, Sepsis‐3 was convened by the SCCM and the European Society of Intensive Care Medicine. At this meeting, sepsis was defined as life‐threatening organ dysfunction caused by a dysregulated host response to infection^18^ (Figure 2). Considering that the sensitivity and specificity for the diagnosis of “SIRS” were not enough to distinguish sepsis from common infections, the SIRS subtype was removed, sepsis and severe sepsis were merged into one subtype (sepsis), and the definition of septic shock remained unchanged.

### 2.2 The evolving concepts of sepsis pathogenesis

Sepsis has always been notorious for being difficult to identify and diagnose. In an effort to better understand sepsis, various theories of its pathogenesis have been proposed over the past century. The earliest and most prevailing theory is that sepsis is caused by an overwhelming inflammatory response to infection. This theory was first proposed by an American physician, William Osler (1849–1919), who suggested that “the patient appears to die from the body's response to an infection rather than from the infection itself.”^19^ This concept was based in part on the observation that no definitive evidence of infection was found in patients presenting with symptoms of sepsis.^20^ This concept was reinforced by Lewis Thomas in a review published in the *New England Journal of Medicine* in 1972, who noted that “the microorganisms…to be rather more like bystanders…. It is our response that makes the disease. Our arsenals for fighting off bacteria are so powerful… that we are more in danger from them than the invaders.”^10^ Since then, the theory that sepsis is a systemic, uncontrolled inflammatory response caused by infection has dominated sepsis research for half a century.

Although Lewis Thomas's theory that the host's own reaction leads to sepsis has become prevalent, investigators began to challenge this theory as early as the 1990s.^21^, ^22^, ^23^ Munford and Pugin proposed that immune cell activation and cytokine secretion are the body's protective mechanisms and that blocking these processes may worsen outcomes.^23^ Weighardt and colleagues^24^ reported that sepsis after surgery was associated with defects in cytokine production, and the survival of septic patients was associated with the recovery of the inflammatory response but not the anti‐inflammatory response. Therefore, immunosuppression is likely to be a primary response to sepsis rather than a compensatory response.^24^ Other investigators have hypothesized that the pathogenesis of sepsis may involve a sequential process from the initial overactivation of the inflammatory response to the subsequent immunosuppression.^21^, ^25^, ^26^

The importance of immunosuppression in sepsis pathogenesis has gradually been recognized. As early as the 1970s, the blood of sepsis patients was reported to contain immunosuppressive factors that might explain the decline in host immunity after thermal injury.^27^ However, in the history of sepsis research, the concept of immunosuppression has been overwhelmed by the concept of an uncontrolled inflammatory response. The role of immunosuppression in sepsis has been reemphasized only over the past decade. In 2013, Hotchkiss et al.^28^ reviewed studies on immunosuppression in sepsis and proposed two theories: in theory 1, he proposed that early death from sepsis is often due to an overactivated inflammatory response that causes a “cytokine storm” response, manifested by fever, refractory shock, acidosis, and hypercatabolism. For a subset of patients, both innate and adaptive immunity are restored, and they survive severe infection. However, if sepsis persists, the patient will enter a state of significant immunosuppression, resulting in the inability to clear primary and secondary infections, which ultimately leads to death. In theory 2, a competing theory, Hotchkiss posited that late mortality in patients with sepsis is still due primarily to persistent underlying innate immune‐driven inflammation, with immunosuppression being a downstream response to inflammation. Although the relationship between the inflammatory response and immunosuppression is still controversial, there is now a consensus that the coexistence of the two may be the core of the pathogenesis of sepsis. In 2016, Sepsis‐3 officially removed SIRS from the diagnostic criteria for sepsis and changed the mechanism that triggers sepsis from an “uncontrolled inflammatory response to infection” to a “dysregulated host response to infection.” Nevertheless, although the concept of a dysregulated host response was proposed at Sepsis‐3, the precise definition and mechanism of this response have not yet been determined. Does a dysregulated host response refer to the conflict between an increased inflammatory response and suppressed adaptive immunity? Do the two occur independently or affect each other? These questions remain to be answered.

Other concepts involved in the pathogenesis of sepsis, including endothelial damage (causing widespread vascular hyperpermeability and tissue edema),^29^ altered mitochondrial function (participating in shaping the inflammatory environment by activating key signaling cascades),^30^ and cell death via apoptosis/pyroptosis/ferroptosis,^31^ are still in the basic research stage and have not yet entered clinical trials. Whether these concepts can be applied clinically depends on subsequent studies with well‐designed preclinical experiments and the use of animal models that can better simulate actual clinical sepsis.

## 3 CLINICAL TRIALS OF SEPSIS DRUGS DEVELOPED ON THE BASIS OF DIFFERENT CONCEPTS

In this section, we review the drugs and clinical trials designed according to different concepts of sepsis pathogenesis. We seek to summarize the factors that led to the failure of historical clinical treatments and the prerequisites for the success of future drugs.

### 3.1 Clinical trials of drugs targeting inflammatory mediators

**FIGURE 3:**
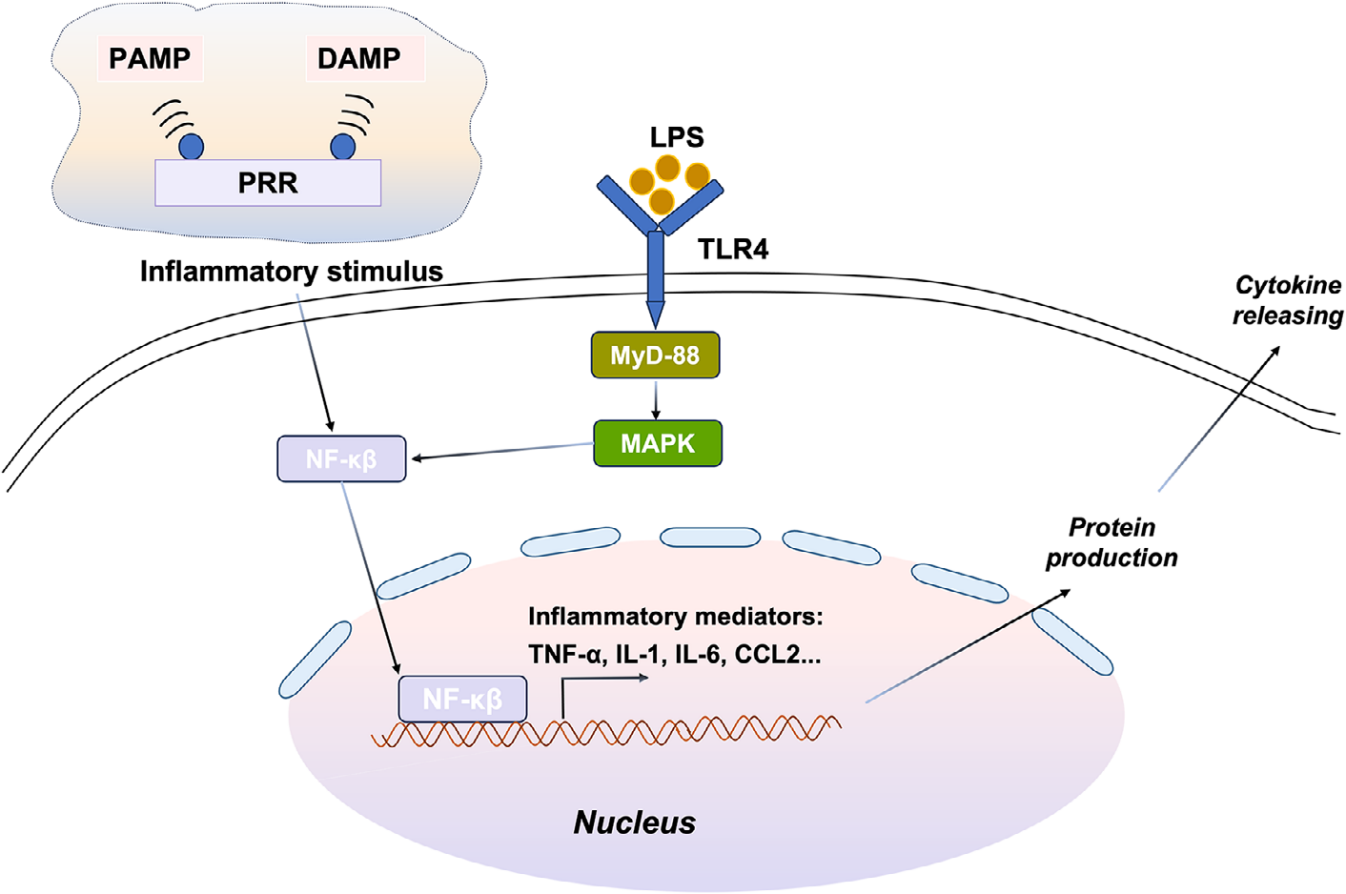
Schematic diagram of the expression of inflammatory mediators induced by pathogen invasion. Liposaccharide (LPS) and Toll‐like receptor 4 (TLR4) are representatives of a concept that pathogens are recognized by a group of receptors called pattern recognition receptors (PRRs) that detect pathogen‐associated molecular patterns (PAMPs) or damage‐associated molecular patterns (DAMPs). After PRRs recongnize PAMPs or DAMPs, the NF‐𝛋B signaling pathway is activated through TLR or NLR, kinase cascades, I𝛋B degradation, and NF‐𝛋B nuclear translocation, resulting in production of inflammatory mediators. This process is considered to be a key mechanism initiating the systemic inflammatory response in sepsis.

According to the inflammation‐centered theory of sepsis pathogenesis, blocking the key mediators of the inflammatory cascade is a logical treatment option. In the 1960s, Otto Westphal proposed that “one of the most important fields of investigation is the search for mediators elicited by endotoxic signals and for the types of cells producing such highly active secondary products, with the hope to finally purify, identify, and even synthesize these biologically most interesting agents.”^32^ Liposaccharide (LPS) and Toll‐like receptor 4 (TLR4) are representatives of a concept proposed by Charles Janeway^33^ that pathogens are recognized by a group of receptors called pattern recognition receptors that detect pathogen‐associated molecular patterns. After recognizing pathogens, innate immune cells release inflammatory mediators, such as TNF‐α, interleukin (IL)‐1, IL‐6, chemokine (C‐C motif) ligand 2 (CCL2), and activated protein C (APC). These inflammatory mediators act on neutrophils, monocytes–macrophages, and endothelial cells, leading to coagulation activation, vasodilation, and endothelial cell leakage, which is considered the pathophysiological basis of organ dysfunction and hypotension during sepsis^20^, ^34^, ^35^, ^36^, ^37^, ^38^ (Figure 3). As a class of broad‐spectrum anti‐inflammatory drugs, glucocorticoids are the earliest and most widely used drugs in the clinical treatment of sepsis.^20^, ^37^, ^39^

#### 3.1.1 LPS‐induced TLR4

LPS‐induced TLR4 signaling results in the production of nitric oxide and the release of proinflammatory cytokines.^40^, ^41^ HA‐1A (nebacumab) is a monoclonal antibody that targets the rough LPS of *Escherichia coli* J5 to treat sepsis caused by gram‐negative bacteria. Because HA‐1A has not been shown to have a significant therapeutic effect and reduce mortality in patients with sepsis, the United States Food and Drug Administration (US FDA) rejected its application in.^42^, ^43^ Eritoran (also known as E5564) is a synthetic lipid A antagonist that can block the binding of LPS and TLR4. Early preclinical in vitro and in vivo laboratory studies have shown that Eritoran can limit the excessive release of inflammatory mediators associated with LPS and increase the rate of survival in sepsis models.^44^ In 2010, a double‐blind, multicenter phase II trial found that patients with severe sepsis in the Eritoran 105 mg group appeared to have reduced mortality (33.3% in the Eritoran group vs. 56.3% in the placebo group, *p *= 0.105).^45^ However, according to a multinational phase III randomized controlled trial (RCT) published in JAMA in 2013 (NCT00334828), the primary endpoint (28‐day all‐cause mortality) did not significantly differ between the groups: 28.1% (366/1304) in the Eritoran group and 26.9% (177/657) in the placebo group. There was also no significant difference in the key secondary endpoint (1‐year all‐cause mortality): 44.1% (290/657) in the Eritoran group and 43.3% (565/1304) in the placebo group.^46^

LPS was once thought to activate TLRs, thereby initiating an inflammatory response to endotoxin. However, although inhibiting LPS is theoretically beneficial, LPS inhibition may lead to unexpected consequences, such as immunosuppression and increased immune recognition thresholds, which may render anti‐LPS therapy ineffective or even worsen the survival of patients with sepsis.

#### 3.1.2 Tumor necrosis factor‐α

TNF‐α was once considered the most promising target for sepsis treatment. A significant increase in cytokines such as TNF‐α is considered a sign of an overactivated inflammatory response. In 1996, a phase II trial of the TNF‐α antibody fragment MAK195F revealed that treatment with different doses of MAK195F did not increase survival in patients with severe sepsis/septic shock.^47^ Adalimumab (Humira) is an anti‐TNF‐α monoclonal antibody approved by the US FDA in 2002 and is widely used to treat autoimmune diseases, including psoriasis, rheumatoid arthritis, ankylosing spondylitis, and inflammatory bowel disease. In 2017, Humira's sales reached $18 billion, making it the world's highest‐selling drug.^48^, ^49^, ^50^ In theory, blocking TNF‐α can inhibit inflammatory responses and may have a therapeutic effect on sepsis. However, the most common adverse reaction to Humira is infection,^50^ and the most serious adverse reactions are severe infection and sepsis.^51^, ^52^, ^53^, ^54^ Sawamura et al.^55^ reported the case of a patient with rheumatoid arthritis who developed severe bacterial sepsis and tuberculous lymphadenitis after treatment with Humira. The theoretical expectations of blocking TNF‐α are inconsistent with or even contrary to the actual clinical results, suggesting that the concept of targeting TNF‐α to treat sepsis may be a dead end and needs to be re‐evaluated and discussed in depth.

#### 3.1.3 Interleukin‐6

IL‐6 is a cytokine with pleiotropic activity^56^ and one of the cytokines with the greatest increase in inflammatory response, which provides a natural advantage in the diagnosis of sepsis.^57^, ^58^ However, the role of IL‐6 in predicting the outcomes of patients with sepsis is controversial. Some studies have revealed that among all inflammatory indicators, IL‐6 can be regarded as an independent risk factor for 28‐day mortality, and it has greater diagnostic and prognostic value for sepsis and septic shock than PTX3 and PCT do.^59^, ^60^ However, according to a study by Yu et al.,^61^ IL‐6 levels did not significantly correlate with death in sepsis patients. Tocilizumab is a monoclonal antibody that targets the receptor of IL‐6 and was approved by the US FDA in 2003 for the treatment of autoimmune diseases, including rheumatoid arthritis, neuromyelitis optica, and giant cell arteritis. Since 2020, tocilizumab has been used extensively in clinical trials for coronavirus disease 2019 (COVID‐19) patients. The efficacy of tocilizumab in patients with COVID‐19 is controversial. Earlier studies reported that tocilizumab can increase the likelihood of survival of COVID‐19 patients. In May 2020, Xu et al.^62^ reported that, compared with conventional treatment, tocilizumab treatment increased the clinical efficacy in 21 patients with severe COVID‐19. In May 2021, RECOVERY Collaborative Group^63^ reported that the mortality rate of COVID‐19 patients who received conventional treatment plus tocilizumab was slightly lower than that of patients who received conventional treatment (31 vs. 35%), and in COVID‐19 patients with hypoxia and systemic inflammation, the use of tocilizumab increased the survival rate and other clinical outcomes. A meta‐analysis of 10,930 COVID‐19 patients revealed that the absolute risk of death with IL‐6 antagonists (including tocilizumab and salidomide, another IL‐6R monoclonal antibody) was 22%, whereas the assumed risk of death was 25% with conventional care or placebo. However, secondary infections occurred within 28 days in 21.9% of patients treated with the IL‐6 antagonist, compared with 17.6% of patients treated with conventional care or placebo.^64^ Another meta‐analysis of 33 clinical trials revealed that tocilizumab treatment significantly increased the risk of fungal infections.^65^ Snow et al.^66^ reported that tocilizumab reduced the need for mechanical ventilation and was associated with benefits in terms of secondary outcomes but did not reduce the intensive care unit (ICU) length of stay. Peng et al.^67^ reported that tocilizumab did not reduce all‐cause mortality, nor did it increase patients' risk of secondary infections or serious adverse events. Overall, these reports have shown that the ability of IL‐6 antagonists to increase the likelihood of survival COVID‐19 patients is questionable, and IL‐6 antagonists should be used with caution because they may increase the risk of secondary infections, particularly fungal infections.

#### 3.1.4 Interleukin‐1

IL‐1 is an important inflammatory mediator. A phase III RCT in which an IL‐1RA antibody was used to treat sepsis was terminated early after interim analysis because no significant difference in mortality was observed between the IL‐1RA group (*n* = 350; mortality 33%) and the placebo group (*n* = 346; mortality 36%).^68^

A Hellenic Sepsis Study Group conducted a phase I trial in which IL‐1RA intervention in sepsis patients resulted in an exaggerated immune response characterized by elevated ferritin levels. The trial revealed improvements in the Sequential Organ Failure Assessment (SOFA) score, alanine aminotransferase level, and international normalized ratio during the first week of treatment. However, the reduction in SOFA scores did not translate into a survival benefit, possibly because of the need for a longer treatment duration.^69^ The group is currently conducting a new phase II clinical trial, NCT04990232. Although the efficacy of IL‐1RA in treating sepsis has not yet been determined, significant efficacy of IL‐1RA in the treatment of autoimmune diseases, such as cryopyrin‐associated periodic syndromes and rheumatoid polyarthritis, has been demonstrated. Anakinra (brand name Kineret) is a recombinant human IL‐1R antagonist (rhIL‐1Ra) that was approved for the treatment of rheumatoid arthritis in November 2001 and was approved for the treatment of cryopyrin‐associated periodic syndromes in January 2013. Currently, the main use of anakinra is to treat autoimmune diseases, including Schnitzler syndrome, systemic juvenile idiopathic arthritis, gout, calcium pyrophosphate deposition, Behçet's disease, ankylosing spondylitis, uveitis, and other autoinflammatory syndromes in addition to rheumatoid arthritis.^70^, ^71^ During the COVID‐19 pandemic, the European Medicines Agency (EMA) and US FDA announced criteria for the use of anakinra to treat hospitalized COVID‐19 patients and those at risk for progression. While initial trials revealed that anakinra may be beneficial in treating COVID‐19, subsequent confirmatory studies failed to replicate the positive data.^72^ On the basis of the results of a clinical trial of anakinra for the treatment of COVID‐19, Shapiro et al.^72^ suggested that the use of the hyperinflammation or cytokine storm paradigm to guide COVID‐19 treatment is unlikely to progress.

#### 3.1.5 Chemokine (C‐C motif) ligand 2

CCL2, also known as monocyte chemoattractant protein 1 (MCP1), is secreted mainly by monocytes/macrophages or dendritic cells and has chemotactic effects on monocytes and basophils. CCL2 signaling through its receptor CCR2 is a key chemotactic signal for monocyte‒macrophage recruitment to tissue lesions.^73^ Drugs that target CCL2 have been used in clinical trials, mostly for cancer treatment,^74^, ^75^, ^76^, ^77^ and some are used to treat autoimmune diseases (e.g., rheumatoid arthritis).^78^ Various agents that target CCL2 and CCR2 have been tested in clinical trials for cancer therapies, but most are ineffective, and several have been abandoned, including carlumab,^79^, ^80^, ^81^ plozalizumab, and PF‐04136309.^82^ Currently, no clinical trials have evaluated the use of CCL2 or CCR2 antagonists for the treatment of sepsis.

#### 3.1.6 Activated protein C

APC is a natural anticoagulant that can proteolytically inactivate coagulation factors Va and VIIIa. Xigris (recombinant human APC) is an anticoagulant drug with anti‐inflammatory effects and was once considered the most promising new drug for treating sepsis.^83^ Xigris completed a phase III clinical trial^84^ and was approved by the US FDA in November 2001. Unfortunately, although initial clinical trials revealed that Xigris was effective in sepsis treatment, subsequent multicenter RCTs failed to confirm that Xigris could increase the survival rate of patients with sepsis, and Xigris was associated with an increased incidence of serious bleeding complications.^85^, ^86^ No benefit of Xigris was found in a clinical trial of children with severe sepsis, and numerically more instances of central nervous system bleeding were observed in the Xigris group than in the placebo group (4.6 vs. 2.1%), particularly in children younger than 60 days.^87^ Finally, on October 25, 2011, the EMA issued an announcement on the withdrawal of Xigris, and Eli Lilly announced the termination of all clinical trials on Xigris.^88^, ^89^ With the failure of Xigris, significant challenges are faced in the application of drugs that target inflammatory mediators in sepsis treatment.

#### 3.1.7 Glucocorticoids

Glucocorticoids can be regarded as broad‐spectrum anti‐inflammatory agents that are used as adjuvant drugs for sepsis and septic shock. The first reported double‐blind clinical trial for sepsis treatment occurred in 1963, when clinical researchers attempted to modulate the inflammatory system with high doses of corticosteroids.^39^ In 1976, Schumer reported that high‐dose methylprednisolone and dexamethasone increased the survival rate of patients with septic shock.^90^ However, whether high‐dose corticosteroids should be used to treat sepsis remains controversial.

In 1987, a double‐blind RCT revealed no benefit from the use of high‐dose corticosteroids for the treatment of severe sepsis and septic shock and that significantly more deaths were associated with secondary infections.^91^ Although high‐dose glucocorticoid therapy has essentially been excluded from the treatment of sepsis because higher cumulative doses and longer treatment durations of steroids have no clear benefit in terms of sepsis survival and are more likely to lead to disseminated fungal infection, avascular necrosis, and osteonecrosis, low‐dose glucocorticoids may be beneficial in the treatment of sepsis.^92^, ^93^, ^94^

In 2018, a large RCT (Adjunctive Corticosteroid Treatment in Critically Ill Patients with Septic Shock, ADRENAL, NCT01448109) involving 3658 patients revealed that continuous infusion of hydrocortisone did not reduce 90‐day mortality compared with placebo in patients with septic shock. In this trial, 511 patients in the hydrocortisone group (27.9%) and 526 patients in the placebo group (28.8%) died within 90 days. No significant differences were found between the treatment and placebo groups in terms of 28‐day mortality, the number of days alive and out of the ICU, the number of days alive and out of the hospital, the rate of recurrence of shock, the rate of patients requiring renal replacement therapy, or the incidence of secondary infections.^95^

A multicenter, double‐blind RCT (NCT00625209) evaluated whether hydrocortisone plus fludrocortisone or with drotrecogin alfa (activated) could improve outcomes in patients with septic shock. Compared with placebo, hydrocortisone plus fludrocortisone significantly reduced patient mortality, with 90‐day mortality rates of 43.0% (264 of 614 patients) and 49.1% (308 of 627 patients), respectively (*p *= 0.03). The incidence of serious adverse events was not significantly different between the groups, but hyperglycemia was more common in the hydrocortisone plus fludrocortisone group.^96^ According to the results of clinical trials of glucocorticoids for the treatment of sepsis to date, short‐term use of low‐dose glucocorticoids may be beneficial depending on the specific conditions of the patients, whereas long‐term use of high‐dose glucocorticoids should be avoided (Table 1).

**TABLE 1:** Clinical trials of drugs targeting inflammatory mediators.

Drugs	Target	Study phase	Conclusion	Sources
Nebacumab (HA‐1A)	LPS	Terminated	Nebacumab did not reduce mortality in patients with sepsis.	1993,^42^ 1991^43^
Eritoran (E5564)	LPS	Phase III	There was no significant difference in 28‐day all‐cause mortality between the Eritoran and placebo groups.	2010,^45^ 2013^46^
Afelimomab (MAK195F)	TNF‐α	Phase II	Afelimomab did not increase survival in patients with sepsis.	1996^47^
Tocilizumab	IL‐6	Phase III	The efficacy of tocilizumab in patients with COVID‐19 is controversial.	2020,^62^ 2021,^63^ 2021,^64^ 2022,^65^ 2022,^66^ 2022^67^
rhIL‐1ra	IL‐1	Phase I/II, phase III	There was no statistically significant reduction in mortality between the IL‐1RA and placebo groups.	1997,^68^ 2022^69^ NCT04990232 (Ongoing)
Xigris	APC	Phase III	Xigris did not increase the survival rate of patients with sepsis but was associated with an increased incidence of serious bleeding complications.	2001,^84^ 2005,^85^ 2007^87^
Glucocorticoids	Broad‐spectrum Anti‐inflammation	Phase III, phase IV	Short‐term use of low‐dose glucocorticoids may be beneficial depending on the specific conditions of the patients, while long‐term use of high‐dose glucocorticoids should be avoided.	1976,^90^ 1987,^91^ 2019,^97^ 2018^96^

Abbreviations: APC, activated protein C; COVID‐19, coronavirus disease 2019.; IL, interleukin; LPS, lipopolysaccharide; TNF‐α, tumor necrosis factor‐α.

### 3.2 Clinical trials of drugs aimed at alleviating immunosuppression

In recent years, the role of adaptive immune suppression in the occurrence and progression of sepsis has been recognized. Strengthening the host's defense against invading pathogens by strengthening the adaptive immune response is also logical. Immune‐boosting therapies for the treatment of sepsis currently in clinical trials have two main mechanisms: broad‐spectrum stimulation, including the administration of immunoglobulins, granulocyte/granulocyte‐macrophage colony‐stimulating factor (G/GM‐CSF), interferon (IFN)‐γ, and thymosin‐α; and direct activation (or blockade) of key molecular targets in the immune activation (or inhibition) pathway, such as IL‐7 administration and programmed death‐1 (PD‐1)/programmed death ligand 1 (PD‐L1) blockade.

#### 3.2.1 Immunoglobulins

Immunoglobulins have been widely used to treat various diseases in neurology, hematology, immunology, nephrology, rheumatology, and dermatology.^98^ The first clinical trial of immunoglobulins for the treatment of sepsis was reported in 1980. Jones et al. reported that the use of immunoglobulins reduced *Pseudomonas aeruginosa* infection and mortality in burn patients.^99^ The ability of immunoglobulins to benefit patients with sepsis has long been controversial. A large retrospective paired study (UMIN‐CTR000012543) revealed that the use of low‐dose immunoglobulin G (IgG) to treat patients with sepsis and septic shock did not reduce mortality.^100^ Hentrich et al.^101^ reported that treatment with immunoglobulins rich in IgM, IgA, and IgG did not reduce mortality in patients with sepsis. Owing to insufficient evidence to support the use of immunoglobulins for the treatment of sepsis, the Surviving Sepsis Campaign guidelines do not recommend the routine use of immunoglobulins for the treatment of sepsis.^102^ Currently, a large‐scale RCT (NCT04182737) is being performed on the use of IgM‐rich immunoglobulin adjuvant therapy, with personalized doses on the basis of serum IgM titers combined with standard doses to treat septic shock.^103^ This study is still ongoing, and its results will help determine the clinical efficacy of IgM‐rich immunoglobulin in reducing the 28‐day all‐cause mortality of patients with sepsis. Immunoglobulins were also tested in clinical trials during the COVID‐19 pandemic. An earlier clinical study of 59 patients with severe COVID‐19 (IRCT20200501047259N1) revealed that immunoglobulin infusions can improve clinical outcomes and reduce mortality in patients with severe COVID‐19 who do not respond to initial treatment.^104^ However, a clinical trial of 84 patients (IRCT20151227025726N20) revealed no difference in mortality between the immunoglobulin treatment group and the control group.^105^ A large clinical trial including 593 participants at 63 sites in 11 countries (NCT04546581) revealed that when combined with standard treatments, including remdesivir, immunoglobulin infusion did not show efficacy in COVID‐19 patients who did not experience end‐organ failure.^106^ A study of 461 participants (NCT04847141) revealed that a subcutaneous injection of immunoglobulin C19‐IG20% was safe for asymptomatic COVID‐19 patients but did not prevent the development of symptomatic COVID‐19.^107^ Overall, immunoglobulin infusion is safe, but conclusive evidence that immunoglobulins have a significant therapeutic effect on sepsis is lacking.

#### 3.2.2 Granulocyte/granulocyte‐macrophage colony‐stimulating factor

G/GM‐CSF exerts immunostimulatory effects by inducing the proliferation and differentiation of various immune cells, such as neutrophils, monocytes, macrophages, and dendritic cells.^108^, ^109^ The earliest clinical trials of G‐CSF and GM‐CSF were conducted in 1994 and 1995, respectively. The results showed that G‐CSF was well tolerated and induced a significant increase in the absolute concentration of neutrophils in the peripheral blood and bone marrow of 42 newborns with presumed bacterial sepsis,^110^ and the administration of GM‐CSF to 20 neonates with extremely low body weights was well tolerated and resulted in significant increases in the neutrophil count, monocyte count, platelet count, and bone marrow neutrophil storage pool.^111^ The effects of G‐CSF and GM‐CSF on increasing the number of granulocytes and monocyte‐macrophages have been widely reported.^110^, ^111^, ^112^, ^113^, ^114^, ^115^, ^116^, ^117^, ^118^ Among clinical trials that include survival analysis, some trials revealed that G‐CSF/GM‐CSF had no significant effect on reducing neonatal mortality,^116^, ^117^, ^119^, ^120^, ^121^ whereas other studies revealed that G‐CSF/GM‐CSF can significantly reduce neonatal sepsis‐related mortality.^115^, ^122^, ^123^ In adult sepsis, Cheng et al.^124^ reported that G‐CSF could not reduce fulminant sepsis caused by melioidosis. A phase II clinical trial of 10 patients with severe sepsis and respiratory dysfunction revealed that GM‐CSF could increase gas exchange without pulmonary neutrophil infiltration.^125^ A clinical trial involving 38 patients with severe sepsis or septic shock revealed that biomarker‐guided GM‐CSF treatment was safe and effective in restoring monocyte immune competence.^118^ A COVID‐19 study revealed that G‐CSF can reduce the number of patients who develop critical illness.^126^ Overall, G‐CSF and GM‐CSF have been confirmed to increase the number and function of leukocytes and reduce the mortality of some neonatal sepsis patients, but more evidence is needed to determine whether these agents can reduce the mortality of adult sepsis patients.

#### 3.2.3 Thymosin α1

Thymosin α1 is a peptide with multiple biological activities and is mainly used clinically to strengthen the immune response.^127^, ^128^ In approximately 2000, thymosin α1 was used to treat hepatitis B virus/hepatitis C virus infections,^129^ human immunodeficiency virus (HIV) infection,^130^, ^131^, ^132^ non‐small cell lung cancer (NSCLC),^133^, ^134^ hepatocellular carcinoma, and malignant melanoma.^135^, ^136^, ^137^ In 2013, a clinical trial of thymosin α1 for the treatment of sepsis involving 361 patients revealed that thymosin α1 could increase the expression of mHLA‐DR and reduce the mortality rate of sepsis (26 vs. 35%).^138^ A multicenter RCT to test the safety and efficacy of thymosin α1 in the treatment of sepsis (NCT02867267) has been completed, but the results have not yet been published. Thymosin α1 has also been used for the treatment of COVID‐19. Two studies revealed that thymosin α1 could reduce the mortality of COVID‐19 patients,^139^, ^140^ whereas other studies revealed no significant differences between the treatment and placebo groups.^141^, ^142^, ^143^, ^144^, ^145^, ^146^ A multicenter cohort study revealed that thymosin α1 use was significantly associated with a higher nonrecovery rate than non‐thymosin α1 use was.^147^ A meta‐analysis revealed that the existing evidence did not support the use of thymosin α1 in hospitalized adult COVID‐19 patients,^148^ whereas findings from another meta‐analysis suggested that thymosin α1 may reduce mortality in patients with moderate to severe COVID‐19.^149^ A recent multicenter RCT found no conclusive evidence that thymosin α1 reduces 28 day mortality in adults with sepsis; nevertheless, it might have beneficial effects in patients aged 60 years and older and those with chronic diseases.^150^

#### 3.2.4 Interferon‐γ

IFN‐γ is a cytokine secreted by immune cells, including T cells, macrophages, and natural killer (NK) cells.^151^, ^152^, ^153^ IFN‐γ treatment has been reported to upregulate mHLA‐DR expression in patients with sepsis and help eliminate pathogenic bacteria.^154^ A clinical trial (NCT01270490) revealed that the immune function of sepsis patients infected with invasive Candida or Aspergillus partially recovered after IFN‐γ treatment.^155^ A clinical trial of 18 adults and two pediatric patients who had received liver transplants revealed that adjunctive immunotherapy with IFN‐γ was well tolerated and improved immune host defense in sepsis‐induced immunosuppression.^156^ A phase II clinical trial (NCT03332225) is currently underway to evaluate the efficacy of recombinant IFN‐γ in restoring immune function, alleviating organ dysfunction, and reducing mortality in patients with sepsis.^69^ A multinational, multicenter phase III clinical trial (NCT01649921) of personalized treatment for sepsis evaluated the ability of IFN‐γ to improve immune function status and reduce secondary infections in patients with sepsis. To date, the results of the above two clinical trials have not been published.

#### 3.2.5 Interleukin‐7

IL‐7 is a γ‐chain antiapoptotic cytokine that is essential for lymphocyte survival and proliferation.^157^, ^158^, ^159^ A double‐blind RCT including 27 patients revealed that IL‐7 treatment was well tolerated, showed no evidence of inducing a hyperinflammatory response, and reversed significant losses of CD4^+^ and CD8^+^ T cells.^160^ A multicenter clinical trial of 21 patients revealed that IL‐7 reversed sepsis‐induced lymphopenia.^161^ Turnbull et al.^162^ reported the case a patient with severe fungal infection who recovered after IL‐7 treatment. A study of IL‐7 for the treatment of COVID‐19 in 2020 that included 25 patients revealed that IL‐7 treatment resulted in the recovery of lymphocyte counts, which were more than twofold greater in the IL‐7 group than in the control group.^163^

#### 3.2.6 PD‐1 and PD‐L1

PD‐1 and PD‐L1 checkpoint inhibitors have led to remarkable progress in the field of cancer immunotherapy. The earliest laboratory study on the potential use of PD‐1/PD‐L1 in the treatment of sepsis was published in 2009; Huang et al.^164^ reported that PD‐1^−/−^ mice had an increased survival rate in a cecal ligation and puncture (CLP)‐induced sepsis model. Many researchers have speculated that blocking PD‐1/PD‐L1 signaling may be a promising approach for treating sepsis, but most of these efforts remain in the basic research stage. A single ascending‐dose, randomized, placebo‐controlled phase Ib trial (NCT02576457) to assess the safety and pharmacokinetics of an anti‐PD‐L1 humanized monoclonal antibody (BMS‐936559: Bristol‐Myers Squibb) was started on December 2, 2015, and reached its primary completion point on March 15, 2017. Twenty‐four patients with sepsis received different doses of BMS‐936559 infusions (10–900 mg; *n* = 20) or placebo (*n* = 4). The study revealed that BMS‐936559 was well tolerated, and no hyperinflammatory response, such as cytokine storm, was observed.

At relatively high doses, BMS‐936559 was associated with a restored immune status (mHLA‐DR level detected by flow cytometry) over 28 days. However, no conclusion can be drawn about whether BMS‐936559 affects survival (deaths at different doses: 10 mg, 2/4; 30 mg, 2/4; 100 mg, 1/4; 300 mg, 1/4; 900 mg, 0/4; placebo, 0/4). The overall mortality was 25%. The status of this trial is marked as “terminated” on clinicaltrials.gov. A phase I/II clinical trial including 13 patients with sepsis registered in Japan (JapicCTI‐173600) revealed that nivolumab, an anti‐PD‐1 antibody approved for cancer treatment, was well tolerated at a dose of 960 mg and increased the expression of the immune status marker HLA‐DR, but the study lacked a placebo‐controlled survival analysis.^165^ A randomized, double‐blind phase 1b study including 31 adult patients with sepsis at 10 US hospital ICUs (NCT02960854) revealed that the administration of nivolumab did not cause unexpected safety issues such as cytokine storms. The authors concluded that further efficacy and safety studies about nivolumab in sepsis treatment are warranted.^166^ Overall, anti‐PD‐1 or anti‐PD‐L1 drugs did not induce cytokine storms in patients with sepsis and were generally safe, but no effect of these drugs in terms of reducing mortality was observed (Table 2).

**TABLE 2:** Clinical trials of drugs aimed at alleviating immunosuppression.

Drugs	Target	Study phase	Conclusion	Sources
Immunoglobulins	IgG∖IgM∖IgA	Phase II, phase III	Immunoglobulin infusion is generally safe, but conclusive evidence to show that immunoglobulins have a significant therapeutic effect on sepsis is lacking.	1980,^99^ 2017,^100^ 2006,^101^ 2021,^103^ 2019,^104^ 2021,^105^ 2022,^106^ 2023^107^
G/GM‐CSF	Neutrophils, Monocyte–macrophages	Phase I/II, phase III	G‐CSF and GM‐CSF have been confirmed to increase the number and function of leukocytes and reduce the mortality of some neonatal sepsis patients, but more evidence is needed to determine whether these agents can reduce the mortality of adult sepsis patients.	1994,^110^ 1995,^111^ 1998,^112^ 2012,^113^ 1999,^114^ 2001,^115^ 2009,^116^ 2013,^117^ 2009,^118^ 1998,^119^ 2001,^120^ 2013,^121^ 2012,^122^ 2012,^123^ 2007,^124^ 2002,^125^ 2021^126^
Thymosin α1	Thymosin α1	Phase III	There is controversy about the therapeutic effect of thymosin α1; two clinical trials found it to be effective, but another six found it to be ineffective. It might have beneficial effects in patients aged 60 and older and those with chronic conditions.	2013,^138^ 2020,^139^ 2022,^140^ 2021,^142^ 2021,^144^ 2021,^145^ 2021,^147^ 2025^150^
IFN‐γ	IFN‐γ	Phase II, phase III	The ability of IFN‐γ to improve immune status and reduce secondary infections in patients with sepsis needs to be further evaluated.	2012,^154^ 2013,^155^ 2019,^156^ NCT03332225 (ongoing), NCT01649921 (not reported)
IL‐7	IL‐7	Phase II	IL‐7 therapy is helpful in the recovery of lymphatic counts in patients with sepsis.	2018,^160^ 2023,^161^ 2020^163^
BMS‐936559	PD‐L1	Phase Ib	BMS‐936559 was well tolerated but no conclusion can be drawn about whether BMS‐936559 affects the sepsis survival rate.	NCT02576457 (terminated)
Nivolumab	PD‐1	Phase I/II	The safety of nivolumab in patients with sepsis has been demonstrated, but an effect in terms of reducing mortality has not been observed.	2020,^165^ 2019^166^

Abbreviations: G/GM‐CSF, granulocyte/granulocyte‐macrophage colony‐stimulating factor; IFN, interferon; IL, interleukin; PD‐1, programmed death‐1; PD‐L1, programmed death ligand 1.

### 3.3 Clinical trials designed on the basis of other concepts

#### 3.3.1 Statins

Statins are a class of drugs, including lovastatin,^167^, ^168^ pravastatin,^169^, ^170^ simvastatin,^171^, ^172^ atorvastatin,^173^ fluvastatin,^174^ rosuvastatin,^175^ and pitavastatin,^176^ that lower blood lipids by inhibiting 3‐hydroxy‐3‐methylglutaryl coenzyme A reductases. The use of statins in the treatment of sepsis is based on the discovery that statins play a role in activating signaling proteins such as small GTPases, which bind to LFA‐1 (CD11a/CD18), thereby affecting leucocyte responses and reducing inflammatory responses in sepsis.^177^, ^178^, ^179^, ^180^, ^181^, ^182^ However, clinical trials have shown that statins have no significant therapeutic effect on sepsis. Chen et al. analyzed 9 clinical trials in which statins were used to treat sepsis in a total of 2,333 patients,^183^, ^184^, ^185^, ^186^, ^187^, ^188^, ^189^, ^190^ and they reported that statins could not reduce mortality in patients with sepsis.^191^ Another systematic review and meta‐analysis that included 14 trials involving 2628 patients^183^, ^184^, ^185^, ^186^, ^187^, ^188^, ^189^, ^190^, ^192^, ^193^, ^194^ revealed that statins are not recommended for the treatment of sepsis.^195^

#### 3.3.2 Mesenchymal stem or stromal cells

Mesenchymal stem or stromal cells (MSCs) constitute an adult cell population with self‐renewal ability. MSCs are multipotent stromal cells that can differentiate into various cell types, including osteoblasts, chondrocytes, myocytes, and adipocytes.^196^, ^197^, ^198^ The concept of MSC treatment for sepsis was based on the findings from preclinical studies^199^, ^200^ showing that MSCs can reduce the inflammatory response^201^, ^202^ and immunomodulatory properties.^203^ Clinical studies have attempted to use MSCs in the treatment of sepsis. For example, a clinical trial of 10 patients (NCT05283317) revealed that adipose‐derived MSC infusions could reduce SOFA scores and 14‐day mortality, but 28‐day mortality did not differ between the treatment group and the control group.^204^ A multicenter, randomized, case‐controlled, double‐blind trial of MSC therapy for COVID‐19 patients with acute renal injury was registered in 2020 (NCT04445220), but its results have not been reported thus far. A phase II RCT at multiple Canadian academic centers was designed to evaluate the safety, clinical efficacy signals, and potential mechanisms of MSCs in the treatment of septic shock (NCT03369275). The trial was completed in 2020, but no report has been published. A phase I trial to determine whether escalating doses of enhanced MSCs are safe and well tolerated in patients with septic shock began in 2020 and ended in January 2024 (NCT04961658), but its results have not yet been reported. A phase II multicenter, double‐blind RCT of umbilical cord cellular immunotherapy for septic shock (NCT05969275) was registered in 2023; the plan is to enroll 296 patients who are admitted to the ICU with septic shock. Treatment consists of injections of 3 × 10^8^ cryopreserved allogeneic umbilical cord‐derived MSCs or placebo over 30 months. The trial is currently recruiting. In summary, more clinical data are needed to determine whether MSCs have a promising future in treating sepsis.

#### 3.3.3 Complements

Complements are now considered a bridge between innate and adaptive immune responses that enable the host to coordinate defenses against pathogen attacks. In 2002, a double‐blind RCT involving 40 participants revealed that complement C1 inhibitors reduced acute kidney injury in patients with severe sepsis or septic shock.^205^ In 2012, an open‐label RCT involving 61 patients with sepsis revealed that treatment with a high‐dose C1‐esterase inhibitor alleviated inflammatory responses and was associated with increased survival in patients with sepsis.^206^ In 2021, a phase II RCT of 72 participants revealed that a monoclonal antibody against C5a (vilobelimab, IFX‐1) was well tolerated in all dose groups, but there were no significant differences in mortality, vasopressor‐free days, or renal replacement therapy‐free days between the treatment and placebo groups.^207^ In 2022, a multinational phase III RCT was conducted at 46 hospitals in eight countries; in this trial, the C5a antibody vilobelimab was used to treat COVID‐19 patients. This multinational RCT revealed that vilobelimab significantly reduced 28‐day all‐cause mortality (hazard ratio = 0.67, 95% confidence interval: 0.48–0.96; *p *= 0.027).^208^ The results of these trials suggest that inhibitors or antibodies targeting complement are promising for the treatment of sepsis and deserve further exploration.

#### 3.3.4 Traditional herbal medicines

Traditional herbal medicines such as Xuebijing, an herbal‐based intravenous preparation, were found to effectively reduce mortality in patients with sepsis in a large RCT (NCT03238742). A total of 1817 patients were included in the study. There was a significant difference in 28‐day mortality between the placebo and Xuebijing groups (26 .1 vs. 18.8%, *p* < 0.001).^209^

In summary, clinical trials aimed at antagonizing inflammatory responses have not achieved the expected therapeutic effects, and major challenges are faced in the application of drugs targeting inflammatory mediators in sepsis treatment. The immune‐boosting treatments are well tolerated and generally safe, and no obvious drug‐induced hypercytokinemia or cytokine storm has been observed. Among nonspecific immunoenhancing drugs, G‐CSF and GM‐CSF have been revealed to have therapeutic effects in some pediatric patients with sepsis, but whether they also work in adult septic patients remains to be verified; thymosin α1 was reported to be effective for the treatment of sepsis in one trial and for the treatment of COVID‐19 in two trials, but no significant therapeutic effect was observed in other studies; immunoglobulins have not been shown to significantly increase the survival rate of patients with sepsis; and IFN‐γ can relieve severe immunosuppression, but whether it can significantly increase sepsis survival awaits the results of ongoing clinical trials. Among specific immune‐enhancing drugs, IL‐7 can significantly increase the number of lymphocytes, but whether it can increase the survival rate needs to be verified. PD‐1/PD‐L1 blockade therapy is considered generally safe, but its ability to reduce mortality in patients with sepsis has not been observed. Regarding treatments designed on the basis of other concepts, statins are not recommended for the treatment of sepsis; a clinical trial of the traditional drug Xuebijing is currently ongoing, and the investigators are reporting that Xuebijing can reduce sepsis mortality; and most trials of MSC therapy are still ongoing. Other conceptual treatment strategies, including vascular repair to correct tissue edema and hypoxia, reducing cell death (necrosis/apoptosis/ferroptosis, etc.), and regulating mitochondrial function and metabolism, are still in the basic research stage, and clinical evidence is needed to determine whether these conceptual therapies can effectively treat sepsis (Table 3).

**TABLE 3:** Clinical trials designed on the basis of other concepts.

Drugs	Target	Study phase	Conclusion	Sources
Statins (lovastatin, pravastatin, simvastatin, atorvastatin, fluvastatin, rosuvastatin, pitavastatin)	HMG‐CoA	Phase II, phase III	Statin therapy has not been shown to be effective in reducing mortality in patients with sepsis and is not recommended for the treatment of sepsis.	2011,^183^ 2011,^192^ 2013,^184^ 2014,^185^ 2009,^186^ 2013,^187^ 2012,^188^ 2014,^189^ 2017,^190^ 2015,^193^ 2016^194^
MSCs	Immunomodulatory	Phase I/II	Most clinical trials on MSCs are ongoing or have been completed, but the results have not been reported; therefore, no conclusion can be drawn.	2022,^204^ NCT04445220 (not reported), NCT03369275 (not reported), NCT04961658 (not reported), NCT05969275 (ongoing)
C1‐inhibitor C1‐esterase inhibitor Vilobelimab	Complement C1 Complement C1 Complement C5a	Phase II, phase III	Inhibitors or antibodies targeting C1/C5a showed significant therapeutic effects in the treatment of sepsis and deserve further exploration.	2002,^205^ 2012,^206^ 2021,^207^ 2022^208^
Xuebijing	Traditional herbal medicines	Phase III	The 28‐day mortality rate significantly differed between the placebo group and the Xuebijing group.	2019^209^

Abbreviation: MSCs, mesenchymal stem cells.

## 4 WHY ANTI‐INFLAMMATORY TREATMENTS FAIL AND WHAT MIGHT SUCCEED

In both sepsis patients and sepsis animal models, the levels of inflammatory mediators, such as TNF‐α, IL‐6, CCL2, and IL‐1, are known to significantly increase when sepsis occurs.^210^, ^211^, ^212^ These inflammatory mediators are widely used to determine the severity of the inflammatory response and sepsis.^213^ Therapies that target these inflammatory factors were once considered promising for curing sepsis; in particular, numerous animal experiments of drugs targeting TNF‐α and IL‐6 have been performed, and these drugs have been reported to have achieved remarkable therapeutic effects. However, all clinical trials targeting these factors have failed to achieve the expected therapeutic effects. Given the continued failure of drugs that target inflammatory mediators over the past decades, some researchers have called for the rejection of the concept that the hyperinflammatory response and “cytokine storm” are the main causes of sepsis.^214^

Sepsis is an acute organ dysfunction syndrome caused by invading pathogens, including bacteria, viruses, fungi and parasites. When invasion occurs, the body's inflammatory response is similar to mobilizing an army to combat the invader. From the perspective of a bystander, more troops present on the line of defense (increased inflammatory mediators in the blood) logically indicate more severe invasion (more severe sepsis). However, from the perspective of a problem solver, the invader cannot be eliminated by destroying the host's own army. The strategy of targeting inflammatory factors involves attempting to eliminate invaders by suppressing the host's armed forces, which is logically untenable. Some researchers regard the inflammatory response as a “necessary evil” because inflammatory mediators activate both the innate and adaptive immune responses.^215^ It is conceivable that during and after a fierce battle (host immunity vs. microorganisms), many traces will remain on the battlefield. The increase in inflammatory factors indicates the intensity of the battle, and the increase in dead cells (including dead immune cells, epithelial cells, endothelial cells, parenchymal cells, etc.) is the debris left on the battlefield. Focusing too much on these battle traces is unlikely to provide much help in analyzing the nature of sepsis; similarly, trying to erase the traces of war rather than eliminating the causes that triggered the war is unlikely to cure sepsis.

The purpose of sepsis treatment should be to eliminate invaders, that is, to eliminate pathogens that invade the host rather than suppress the body's own defenses. Antibiotics directly target pathogens and are the most direct and effective treatment for sepsis. Before the use of antibiotics, infectious diseases, particularly neonatal infection, puerperal fever, and posttrauma and postoperative infections, were the leading causes of human death. The discovery of penicillin in the 1940s was a milestone in increasing the survival rate of patients with infectious diseases, including sepsis. With the discovery and extensive use of antibiotics, patient mortality has been greatly reduced, and the average life expectancy has significantly increased.^216^, ^217^ The child mortality rate in the United States decreased by more than 90%, and the average life expectancy of the overall population increased by more than 10 years from 1938 to 1956. Demographers call this period “the major turning point in the mortality transition.” To date, early antibiotic intervention (within 1 h after diagnosis) is still emphasized in the treatment of sepsis.^218^ A joint study by 21 emergency departments in the United States revealed that the median time from diagnosis to antibiotic administration was 2.1 h. For every 1‐h delay in antibiotic administration, the mortality rate of sepsis patients increased by 0.3%, and the mortality rate of septic shock patients increased by 1.8%.^219^

However, after the large‐scale use of antibiotics, several important issues began to emerge. The emergence of drug‐resistant bacteria has limited the effectiveness of antibiotics.^220^, ^221^, ^222^ According to the WHO, bacterial antimicrobial resistance directly caused at least 1.27 million deaths and was one of the causes of another 4.95 million deaths in 2019.^223^ Moreover, even if an antibiotic effectively kills sensitive pathogens, the host immune system still needs to be effectively mobilized to eliminate the remaining pathogens. Hosts with immunodeficiency, such as HIV patients (CD4^+^ T‐cell deficiency) and organ transplant patients receiving immunosuppressive drugs, have long been recognized to be more susceptible to fatal infection. For example, even the most advanced broad‐spectrum antibiotics cannot eliminate pathogens in patients with sepsis in the late stage of HIV infection. Therefore, while antibiotics are administered to treat sepsis, the immune system still needs to be properly activated rather than suppressed to improve treatment effects. Notably, low‐dose and short‐term glucocorticoid treatment has shown benefits in some patients with severe acute exudation and acute lung injury who require respiratory support. These findings suggest that at specific stages, especially in the early and acute lung exudation stages, the inflammatory response that causes acute respiratory dysfunction needs to be properly controlled. However, from the perspective of eliminating the pathogens that cause sepsis, strengthening the body's defense force to overpower the invasion force of pathogens may be the correct direction to achieve the goal of curing sepsis.

Several attempts to strengthen host immune responses, including nonspecific immunopotentiators, such as immunoglobulins, G‐CSF/GM‐CSF, IFN‐γ, and thymosin, and immunotherapies directed against specific targets, such as PD‐1/PD‐L1 antibodies, have entered clinical trials, as described above. Notably, “failed” anti‐inflammatory treatments fundamentally differ from “not yet successful” immunotherapies. The former suppresses the host's immune response, which is inconsistent with the logic of eliminating pathogenic invaders; the latter is “theoretically logical,” but discovering the correct strategy to effectively stimulate immune responses takes time.^224^, ^225^, ^226^ To date, the greatest number of clinical trials have used G/GM‐CSF for the treatment of sepsis.^110^, ^111^, ^112^, ^113^, ^114^, ^115^, ^116^, ^117^, ^118^, ^119^, ^120^, ^121^, ^122^, ^123^, ^124^, ^125^, ^126^ The partial success of G/GM‐CSF treatments, especially in neonates with sepsis, suggests that many experimental therapeutic strategies in the laboratory that aim to reduce neutrophil/macrophage numbers or suppress their functions (especially in animal models of ARDS) are likely going in the wrong direction.

In recent years, therapies targeting immune checkpoints (including PD‐1/PD‐L1, CTLA4, etc.) have been widely used in cancer immunotherapy and have changed the treatment landscape of cancers. However, despite the potential curative survival benefit, only a small percentage of patients respond positively to PD‐1/PD‐L1 blockade therapy. Clinical data show that even for patients with highly PD‐L1‐positive tumors, more than 50% may not respond to PD‐L1/PD‐L1 blockade.^227^ The objective response rate is 30–45% in patients with melanoma,^228^ 15–20% in patients with NSCLC,^229^ 13% in patients with head and neck carcinoma,^230^ and 22–25% in patients with kidney cancer.^231^ Although tumor PD‐L1 expression greater than 1% can be considered positive, the expression level of PD‐L1 is still an important factor affecting the efficacy of tumor treatment. The objective response rate of patients with PD‐L1‐positive tumors was significantly greater than that of patients with PD‐L1‐negative tumors, and both overall survival (OS) and progression‐free survival increased.^229^, ^232^ Atezolizumab, an anti‐PD‐L1 antibody, increased OS in the PD‐L1‐positive subgroup of NSCLC patients, with a more pronounced survival benefit observed in patients with higher PD‐L1 expression.^233^

**FIGURE 4:**
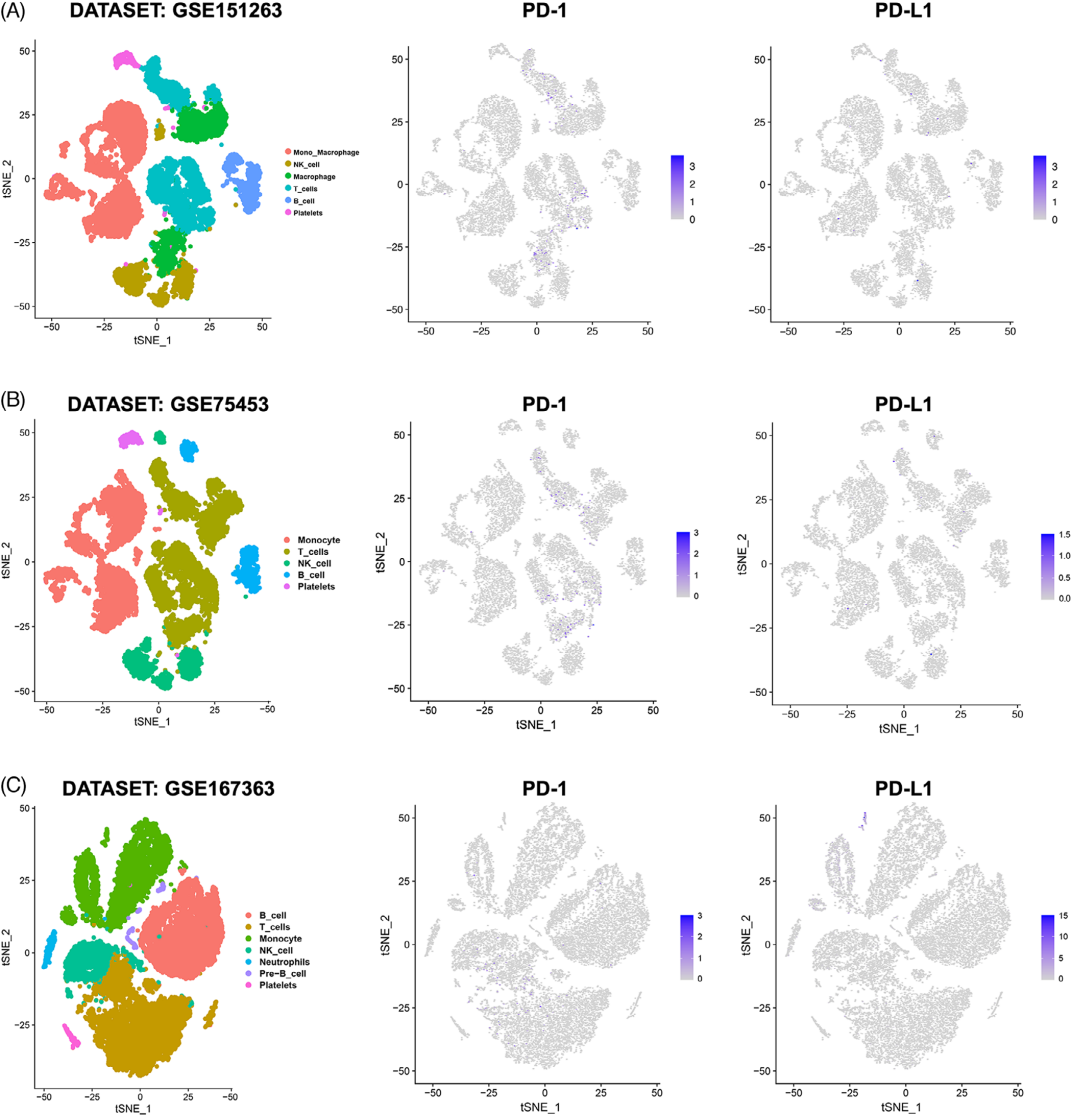
The expression patterns of PD‐1 and PD‐L1 in the peripheral blood mononuclear cells (PBMCs) of septic patients. Single‐cell RNA sequencing (scRNA‐Seq) data in public datasets were analyzed. The percentages of programmed death‐1 (PD‐1)/PD‐1 ligand (PD‐L1)‐positive cells in the PBMCs of septic patients in the three datasets, (A) GSE151263, (B) GSE75453, and (C) GSE167363, were extremely small, accounting for approximately 0.1–0.3% of the total PBMCs.

We hypothesized that blocking specific immunosuppressive targets, such as PD‐1/PD‐L1, has not achieved the expected success because the positive cell ratio of PD‐1/PD‐L1 in the peripheral blood immune cells of septic patients is low. Analysis of single‐cell transcriptome sequencing (scRNA‐Seq) data in public datasets (GSE151263, GSE75453, and GSE167363) revealed that the number of PD‐1/PD‐L1‐positive cells among the peripheral blood mononuclear cells (PBMCs) of patients with sepsis was extremely small; the absolute number of PD‐1‐ or PD‐L1‐positive cells detected in the PBMCs of septic patients was usually 10–30 per 10,000 cells, with a ratio of approximately 0.1–0.3% (Figure 4). Such a low PD‐1/PD‐L1 positive rate is unlikely to cause the widespread and severe immunosuppression observed in sepsis. Even if PD‐1 or PD‐L1 is effectively inhibited, it is difficult to achieve the goal of activating the immune response.

In addition, multiple studies have shown that tumor mutation burden is positively correlated with neoantigen burden and response to immunotherapy.^234^, ^235^ Tumors with high mutation loads are more sensitive to PD‐1/PD‐L1 treatment, whereas some tumors with low mutation loads, such as pancreatic cancer, are usually ineffective at blocking PD‐1/PD‐L1.^236^ Infection is an acute event in the body, whereas tumorigenesis is a relatively chronic event, and the mechanisms of immunosuppression in the two greatly differ. Tumor immune escape is more about the inability of immune cells to correctly identify and kill malignant cells. However, during infection, gene mutations usually do not occur. Sepsis immunosuppression may involve natural reactions, such as anti‐inflammatory compensatory reactions, the emergence of myeloid‐derived suppressor cells (MDSCs), and lymphocyte apoptosis, during the evolutionary process. A deeper analysis of immunosuppression in sepsis is needed to develop more precise and effective immune‐enhancing treatments.

Other immunosuppressive molecules, such as CTLA‐4,^237^ TIM3,^238^ and LAG3,^239^ have been tested in animal models, but none have entered clinical trials for sepsis treatment. For treatments that target these immunosuppressive molecules to be successful, whether they in fact mediate immunosuppression in sepsis needs to be verified. The abundance of these genes in immune cells, whether their expression changes during sepsis, and whether the absolute number of cells expressing them changes during sepsis (rather than just a change in the relative proportion of cells) are prerequisites for their success. Simply shifting tumor immunosuppression targets to the treatment of sepsis is unlikely to easily achieve success.

## 5 FUNDAMENTAL PRINCIPLES OF PRECLINICAL STUDIES TO ENSURE SUCCESSFUL CLINICAL TRANSLATION

Whether it is a new treatment based on the principle of strengthening the host's defense forces, a new therapeutic strategy designed on the basis of a newly emerging concept, a new target discovered through bioinformatic analysis based on the “black box” principle, or even a traditional herbal medicine—all avenues of treatment should be viewed with an open mind, but all new treatments must be rigorously verified in well‐designed preclinical studies. The fundamental principles of preclinical studies should be simplified to ensure successful clinical translation:

First, each animal sepsis model should simulate a specific clinical event as closely as possible under the current technical conditions. For example, CLP is used to simulate severe abdominal infection caused by intestinal perforation,^240^, ^241^, ^242^ and airway intubation and injection of bacteria or viruses can be used to simulate pneumonia caused by pathogenic microorganisms.^243^, ^244^, ^245^, ^246^, ^247^, ^248^, ^249^ Animal models that are completely different from clinical reality, such as those involving LPS injection, should no longer be used in sepsis studies. As early as 2003, investigators reported that LPS injection may serve as a model for endotoxic shock but not sepsis.^20^ The commonly used doses of LPS (5–25 mg/kg) result in blood concentrations (50–250 µg/mL) in mice that are approximately 10^4^–10^5^ times higher than the median endotoxin level in the blood of patients with sepsis (100–700 pg/mL). In addition, unlike microorganisms, LPS cannot proliferate or replicate continuously in the host and elicit a sustained immune response, which makes translating the results obtained from LPS models to clinical use almost impossible.

Second, the primary goal of a novel treatment should be to increase the survival rate of septic animals, and the secondary goal should be to reduce the pathogen load in the blood and alleviate the degree of organ injury. A reduction in inflammatory mediators is not sufficient to assess the effectiveness of a therapy, but such a reduction, especially a reduction in TNF‐α and IL‐6 levels, is often used as an independent indicator of improvement in sepsis patients. Some preclinical studies do not include animal survival experiments, and the effectiveness of treatment is judged only on the basis of the level of inflammatory factors measured in vitro. The levels of inflammatory mediators, especially the expression level of TNF‐α, are often not significantly related to the severity of sepsis, and blocking TNF‐α may worsen sepsis severity or lead to secondary infection, which suggests that inflammatory factors such as TNF‐α may not be suitable indicators for assessing the severity of sepsis and whether symptoms of sepsis are alleviated.

Third, the number of animals used in survival experiments must be sufficient. Some opinions on animal ethics suggest that the number of mice in each group should be limited (for example, no more than 10 mice per group) and that therapeutic experiments should not be repeated multiple times, which is considered to violate animal welfare ethics. However, sepsis is a notoriously heterogeneous syndrome. Animal models, such as the CLP model, also generate considerable heterogeneity in terms of severity. If the number of mice in each group is insufficient (e.g., *n* = 10 or less), the assessment of the therapeutic effect will be significantly affected by heterogeneity because each death/survival will cause a 10% change in mortality. In addition, assessing the actual effect of an intervention requires biological replicates; otherwise, determining whether a positive or negative result is due to the intervention or simply the heterogeneity of the animal model will be difficult. Reducing the number of animals used is indeed an aspect of animal welfare. However, a sample size that is too small and a lack of sufficient repetitions are likely to distort the actual effect of an intervention, which hinders the objective assessment of the actual therapeutic effects of an intervention. Thus, if the sample size is reduced simply for the sake of ideal animal ethics, more animals will be used later to validate the initial intervention, which will in turn significantly increase the number of animals actually used.

On the basis of the above principles, to further ensure the success of translational research from the bench to the bedside, factors such as optimization of drug doses, selection of different time windows for administration, and monitoring of adverse reactions should also be considered. In addition, according to a consensus meeting on animal sepsis models held in Vienna in 2017, several principles were recommended to improve the quality of preclinical studies,^250^ including that therapeutic interventions should be initiated after the septic insult, that the treatments should be randomized and blinded when feasible, that the microorganisms used in animal models should replicate those commonly found in human sepsis, that sepsis models could be initiated at sites other than the peritoneal cavity, and that the clinical management used in septic patients, such as antibiotic administration and fluid resuscitation, should be implemented. Trying to meet these conditions will greatly increase the possibility of successful clinical translation.

## 6 CONCLUSION AND PROSPECTS

In 1972, Lewis Thomas first proposed in the *New England Journal of Medicine* that “it is our response that makes the disease…,” suggesting that patients with sepsis are more endangered by the body's own response than by invading microorganisms.^10^ Since then, strategies that target inflammatory and immune mediators have dominated sepsis research for approximately half a century. Perhaps the time has come for new perspectives. Inhibitors of inflammatory mediators, including TNF‐α, IL‐6, and IL‐1 antagonists, are widely used to treat autoimmune diseases and have achieved significant therapeutic effects, but all have failed in clinical trials for the treatment of sepsis, which in itself suggests that inflammation is indeed involved in the pathogenesis of autoimmune diseases but is very unlikely to be the cause of sepsis.

An individual may develop dozens to hundreds of common infections (colds, tonsillitis, gastroenteritis, wound infections, etc.) in his or her lifetime but generally will not develop sepsis. The immune system established during the long evolutionary process can eliminate most invading microorganisms, mainly through innate immune activation (increased body temperature, activation of neutrophils and macrophages to eliminate pathogens, secretion of inflammatory factors, etc.) and the adaptive immune response (T/B lymphocytes recognizing and specifically killing pathogens). Sepsis occurs only when the balance is disrupted and the pathogen force overwhelms the host's defense force. An excessive inflammatory response can be understood as the host's inability to eliminate invading pathogens even if the innate immune response is mobilized to the extreme. This excessive inflammatory response is a reflection of a fierce battle but should not be understood as “it is our own response that causes the disease.” Neither hyperinflammatory responses nor progressive suppression of adaptive immunity should be considered the cause of death in patients with sepsis; rather, death should be considered a consequence of the host's inability to eliminate pathogens.

The pathogenesis of sepsis may not be that complicated. From a mechanistic point of view, sepsis may simply be the consequence of a failing battle against microorganisms when the force of the invading pathogen overpowers the body' defense forces, including both external assisting forces (accurate pathogen identification and sensitive antimicrobial drugs) and internal forces of the host (innate and adaptive immune responses). Regardless of whether the defense force is less than or equal to the invasion force, the host cannot completely eliminate invading pathogens and therefore cannot recover. Therefore, the strategy for treating sepsis should be to mobilize all defense forces to combat invading pathogens. Accurate pathogen identification and the use of the corresponding sensitive antimicrobial drugs are external forces that assist the host in killing pathogens as much as possible. Moreover, more effective activation of the host immune system, especially activation of adaptive immune responses by increasing the number and function of T cells and B cells and fine‐tuning the functions of innate immune cells, including neutrophils, macrophages, and MDSCs, is an internal force that strengthens the host's defense capabilities. Only when the superposition of external assistance and internal immune forces overpowers invading pathogens can sepsis be cured. Future treatment strategies should be focused on strengthening external and internal forces instead of being limited by the concept of anti‐inflammatory treatment.

More than 100,000 papers have been published in the field of sepsis, in which hundreds of treatments and biomarkers have been proposed to have dramatic therapeutic effects on sepsis models.^251^, ^252^, ^253^, ^254^ However, to date, the only proven effective clinical treatments are antimicrobial drugs^255^, ^256^ and life support treatments. The gap between this “paper universe” and clinical reality is so large that we must reevaluate our previous concepts about sepsis. The trend of recent studies seems to be that as long as there are complex signaling pathway analyses and many molecular mechanisms, there is no need to establish animal models that can reflect real clinical scenarios, intervention experiments, or survival analysis. Only a small proportion of laboratory studies can cover these simple principles at the same time. Some studies included intervention experiments but used an LPS‐injection model; some used a CLP model for intervention experiments, but the sample size of each group was too small; and some tested changes in inflammatory mediators but did not include intervention experiments or survival analysis. Notably, almost no papers specifically mentioned that the intervention experiments had biological replicates, as if this was something unimportant and not worth mentioning. Studies that address complex molecular mechanisms but lack appropriate animal models, intervention experiments and survival analyses are unlikely to help us solve actual clinical problems. We believe that if a study can cover these three simple principles, that is, a good animal model, a good intervention experiment, and a sufficient n‐number, even without more complex quality controls such as dose escalation, adjustment of the administration method and timing, and randomized and double‐blind grouping, it has at least taken the first step toward successful clinical translation.

## AUTHOR CONTRIBUTIONS

Wei Zhang, Fei Xiao, and Yan Kang conceived the initial concept. Zhongxue Feng and Lijun Wang performed the literature search and wrote part of the draft. Zhongxue Feng, Tingting Li, and Wei Zhang prepared the figures, and Jing Yang prepared the tables. Yan Kang and Xuelian Liao offered opinions to improve this work. Wei Zhang wrote and revised the paper. All the authors have read and approved the final manuscript.

## CONFLICT OF INTEREST STATEMENT

The authors declare no conflicts of interest.

## ETHICS STATEMENT

Not applicable.

## Data Availability

Not applicable.
